# Dialects, motivation, and English proficiency: Empirical evidence from China

**DOI:** 10.3389/fpsyg.2022.999345

**Published:** 2022-09-29

**Authors:** Rob Kim Marjerison, Shuo Yang

**Affiliations:** ^1^Global Business, College of Business and Public Management, Wenzhou-Kean University, Wenzhou, China; ^2^English in Global Settings, College of Liberal Arts, Wenzhou-Kean University, Wenzhou, China

**Keywords:** language acquisition, motivation, TESOL, social change through applied linguistics, English medium education, dialects, socioeconomic mobility, social sustainability

## Abstract

Within the context of China, this study seeks to examine the relationship between English language proficiency, the native dialect of the learner, and the learner’s reason, or motivation for learning English. English language proficiency can be an important vehicle for accessing high quality higher education, for interacting with non-Chinese, and for enhancing employment and career opportunities Data was gathered through an online survey with 985 usable responses recorded. Respondents included a distribution of speakers from five of the major distinct dialects of China. The analysis provides empirical evidence of a diversity of propensities and motivations for English language acquisition among learners from different regions and native dialects. Access to international higher education as a type of motivation is found to have a moderating effect on English proficiency. Other findings suggest that learners in regions with more historic exposure to foreign interaction are more likely to be motivated for social reasons, those from regions with export focused commerce will be motivated for business related reasons. The results of this study may be of interest to policy makers, linguists, educators, and those with an interest in socioeconomic sustainability through language acquisition and education as a method of socioeconomic mobility.

## Introduction

The role of education in socioeconomic mobility is well established in the existing literature (Tilak, 2010; Cremin and Nakabugo, 2012). English is considered the lingua franca of international commerce (Komori-Glatz, 2018) as well as science (Suzina, 2021), medicine (Tweedie and Johnson, 2019), and, importantly, technology and the Internet (Melitz, 2018). People in many countries, including China, can realize increased opportunities for education and employment as a result of achieving even a modest level of proficiency in English (Curdt-Christiansen and Wang, 2018; Gao and Zheng, 2019). In China, the opportunities are significant, particularly for access to education abroad (Brown et al., 2013; Dimova et al., 2015; Zhang, 2018).

English language study in China has been compulsory for several years with limited results (Reynolds and Teng, 2019). Reasons include inconsistent application of language teaching methods and access to language education as a result of differing regional levels and types of economic development (Cheng, 2009; Zhang, 2018). Some cities have experienced significant numbers of foreigners through tourism or export-related commerce, while other areas focused on agriculture, or upstream and OEM manufacturing (Wei et al., 2007; Yan, 2018) have less incentive to prioritize English language education. Some cities, like Shanghai, have had centuries of interaction with foreigners resulting in a well-established English speaking population (Maclellan, 1889).

Education in China is very competitive, and many high-achieving students seek to study abroad as a way to improve their socioeconomic situations (Tsang, 2013); consequently, the desire of a large number of Chinese students to improve their English test scores is the basis for significant private educational enterprises (Zheng et al., 2021), and despite many decades of English language education in China, there remains a lack of consensus regarding the most effective way to teach English (Wang and Fan, 2020), or more specifically, to improve test scores. Nearly all Chinese speak more than one distinct dialect, and typically, this includes the local or regional dialect of their home city and Mandarin.

This study explores how students’ primary or first Chinese dialect, their most exposed dialect (MED), can affect their perceptions, motivations, and ultimately their level of proficiency in English. Students from five different Chinese regional dialects, including the most predominant dialects of the cities of Shanghai, Wenzhou, Beijing, Shandong, and Guangdong, participated in this study via an anonymous online survey. These five cities were selected from the dozens of major cities in China to provide a diversity of geographical location, size, and industrial base. The survey resulted in 985 useable responses with a reasonable distribution among the five target demographics, that is, speakers of the five dialects considered.

Notably, there is not complete consensus on the distinction between a language and a dialect (Jiang-hua, 2020; Wichmann, 2020), particularly in the case of China. For purposes of this study, and consistent with the accepted view within China, the various forms of spoken communication in China will collectively be referred to as Dialects of the Chinese language. The rationale for this is that while many of these dialects are mutually unintelligible, they share a common written language.

Mapping the ecology of learners’ literacy may assist in the advancement of theories on how native speakers of particular dialects learn and ultimately become proficient in English, and by doing so, better enable educators to provide the learning tools and strategies best suited to enable students from diverse and less affluent backgrounds and areas the opportunities to access the type of education that can help elevate the socioeconomic status of themselves, and their families.

For purposes of this study, English learners in China would learn English as their L2 following the acquisition of either one or two dialects of Chinese (L1).

Given the number of dialects in China, the importance of English proficiency in social mobility, and the complexity of language acquisition, an exploration of variance in Chinese students’ perception, motivation, and proficiency of English from region to region, and the moderating effect of motivation between them, becomes a topic worthy of exploration. A review of the existing literature reveals an area of relative under exploration on this topic. It is this gap, with a focus on differences among most exposed dialects speakers’ motivation for acquisition, and proficiency of English that this study will seek to address. Therefore, the research questions (RQs) of this study are:

Does the native dialect affect learners’ proficiency in English? If so, why?

Does the native dialect affect learners’ (reason) motivation to learn English? If so, why?

## Literature review

### Socioeconomic mobility in China

Since the economic reforms of the 1980s, China has experienced unprecedented commercial development officially lifting the population out of poverty by 2020. However, much of the result has been concentrated in the Eastern coastline and other large metropolitan areas. This study seeks to examine one of the principal drivers of socioeconomic mobility in China, access to international and foreign education, to which the primary hurdle is English language proficiency.

Previous studies have established that English language proficiency is a necessary prerequisite to international education (Marjerison et al., 2020) and is an essential driver of social mobility in China (Mok, 2016; Marginson, 2018; Mok and Marginson, 2021). Access to education plays an essential role in the sustainable development of China’s socioeconomic structure (Mok and Neubauer, 2016). Specifically, with the recognition of highly ranked graduate programs of study, mostly in the US and UK, and the improved career prospects that such an education provides (Jian, 2012; Brown et al., 2013), it has become highly desirable for Chinese students to study abroad at the top universities and return to China after graduation. One of the requirements for overseas students is a high score on English tests, such as TOEFL and IELTS. It can be seen that Chinese students’ perception of English is an essential factor in access to education is a strong motivational factor in learning English (Liu and Chiang, 2019; Zheng et al., 2019).

Participation in the emerging technology-driven economic growth in China requires up-to-date technical skills and training and well-developed creative cognitive abilities, global perspectives, and cross-cultural communications and critical thinking skills (Bi et al., 2019; Atherton et al., 2021). It is through education that interclass mobility is most likely to be achieved in China, and much of that will be driven by international education (Hua, 2019; Wang and Liu, 2019). In short, access to high-quality education, including that which will help learners develop the ability to communicate and collaborate with non-Chinese and ultimately improve their career prospects.

Therefore, students, as well as educators and policy makers, need to explore strategies to help learners achieve greater English language proficiency and higher scores on the TOEFL test (Marjerison et al., 2020), which will enable access to international higher education. English language proficiency is a precursor to accessibility to higher education and the resulting socioeconomic mobility for many young Chinese. As a result of the importance of English language proficiency, learners’ motivation to learn English, whether for educational purposes or to improve career prospects, is an area of interest.

### Chinese dialects – regions

Dialect refers to a “regionally or socially distinctive variety of a language, identified by a particular set of words and grammatical structures” (Watson, 2013). Dialects of different regions of China can be affected by geographical environment and economic conditions, embodied in pronunciation, tone, and slang (Dong, 2020). The considerable linguistic differences among these five, as well as other dialects of China is well documented (Li, 1973; Hsu, 1994; Wang, 2015). The extent and nature of the differences are an important factor in the examination of how native speakers of the dialects may differ in their propensities and, ultimately, their proficiency in English.

The Beijing dialect and Mandarin are similar but not identical. Cantonese and Shanghainese are the most widely spoken dialects in their respective regions of China and are embedded in those regions’ geographical environments and economic development.

For example, in China, the national dialect, Mandarin, and the Beijing dialect(s), known as Putong hua (Zhang and Cai, 2021), share many similarities in sentence structures and pronunciation. The significant difference is that the Beijing dialect emphasizes the rhotic accent. Speakers of the Beijing dialect prefer to add a rhotic accent to every sentence’s last word.

The history of Chinese dialects is complex and complicated due to the large geographic area and the long history of populated areas during which time people did not travel widely nor frequently, and as a result, many dialects developed over time. The sociolinguistic environment in China, where many variations are exhibited in a common culture and writing system is a broad area to explore. How the dialects affect the acquisition of other dialects/languages is relatively under-explored and well worth further examination (List et al., 2014; Tang, 2017).

A thorough exploration of the dialects of China is both outside the scope of this study and beyond what is appropriate for a single study both in scope and magnitude; however, the relevance of promoting multilingualism as a consequence of globalization has been demonstrated (Feng and Kim, 2022), including within the context of nations with many distinct languages/dialects.

**FIGURE 1:**
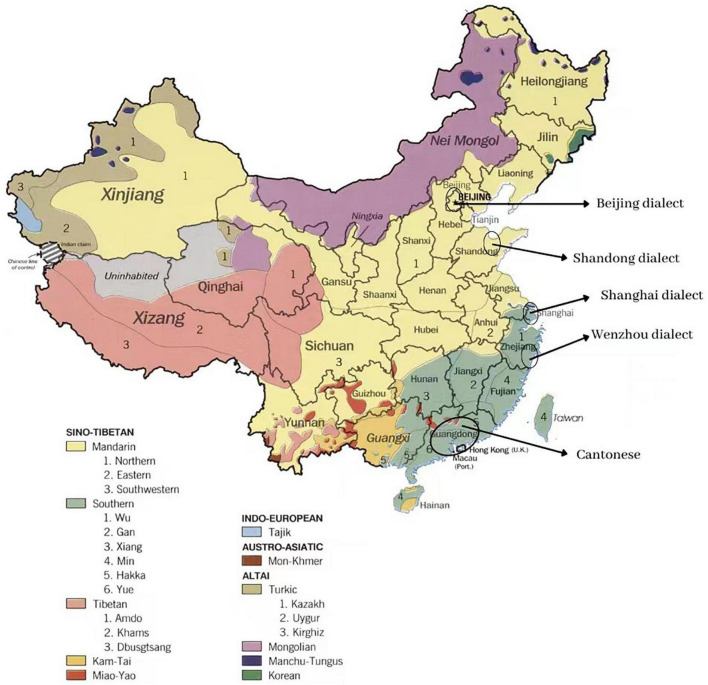
Regional dialects included in this study. Adapted from Wikipedia “Languages of China.” Public Domain. Available at https://en.wikipedia.org/w/index.php?title=Languages_of_China&oldid=1107872021#/media/File:China_linguistic_map.jpg.

The dialects included in this study are most widely spoken in the regions indicated in Figure 1. The Beijing dialect, known as Pekingese or Putong hua, is the most widespread dialect spoken in the capital city of Beijing and the surrounding area. The pronunciation of the Beijing dialect is similar to but not the same as Mandarin. The Beijing dialect is one of the many northern dialects and is considered the standard (Song and Zhu, 2016).

The Shandong dialect is spoken in Shandong’s northern province. The inverted sentence is one of the characteristics of the Shandong dialect, and this kind of inverted sentence is composed of inverting the main sentence and transposition of the last sentence (Tang, 2017; Aaowah, 2019; Dong, 2020).

The Guangdong dialect is also known as Cantonese. Cantonese has more than 2,200 years of recorded history, and it is one of the ten major Chinese dialects in China (Li, 1973; Erbaugh, 1995). The Cantonese dialect is different from other regional dialects and Mandarin especially for its unique linguistic features such as pronunciation, expression, and slang (Lu et al., 2019). For example, in the phonological aspect, Mandarin has four lexical tones, while Cantonese has nine (Ma et al., 2006). In addition, Cantonese has a very different intonation pattern from Mandarin as a result, speakers of dialects in other regions of China have difficulty understanding Cantonese. The global Cantonese-speaking population is significant and stable at about 70 million of whom 40 million live in Guangdong Province, especially in the Pearl River Delta region, making Cantonese an important regional dialect in China (Lu et al., 2019). In particular, the popularity of Cantonese songs, movies, and TV shows in mainland China in the 1990s, known as “Cantonese fever,” also helped spread and popularize Cantonese in other parts of the country (Lu et al., 2019).

Located on China’s southeastern coast, Wenzhou was the first of several “special economic zones” and is well known for its high level of commercial development, the number of Wenzhounese people who have traveled and lived abroad, the uniqueness of the dialect, and for being the country’s largest urban Christian center, hence, being known as the “Jerusalem of China” (Cao, 2010). The Wenzhou dialect is a southern dialect in China and is only spoken in Wenzhou (Newman et al., 2007). The Wenzhou dialect is one of the most unique dialects in China and is impossible for speakers of other languages or dialects to understand. The Wenzhounese tend to build connections within their communities, and their strong sense of ownership has fostered a high level of economic development in light manufacturing and small and medium-sized business (SMEs) (Wei et al., 2007).

“Shanghai Chinese is a Wu dialect spoken in the city of Shanghai, one of the four municipalities in the People’s Republic of China” (Chen and Gussenhoven, 2015). During the 20th century, the Shanghai dialect was heavily influenced by neighboring regions, such as the Jianghuai Mandarin of Zhejiang Province and Jiangsu Province, the Suzhou Wu dialect of nearby Suzhou, and the Wu and Ningbo dialects (Xiao-Quan, 2001). In addition, dialects from more distant areas, Cantonese from the South, and Northern Mandarin, also profoundly influenced the Shanghai dialect. Most Shanghainese native speakers are descendants of immigrants who moved to Shanghai from Jiangsu and Zhejiang provinces in the late 19th and early 20th centuries (Chen and Gussenhoven, 2015).

### Language acquisition

The existing literature on language acquisition is abundant, and while differing theories on learning abound, there is some consensus on the relative ease of language acquisition by learners who already possess proficiency in multiple languages (Sokolova and Plisov, 2019; Hoppe et al., 2020). There is also evidence to support the relationship between the relative complexity of language acquisition of languages within the same linguistic family (Stein-Smith, 2018; Melo-Pfeifer, 2021), and the important role of teaching and pedagogical styles has been well documented (Zhang and Sun, 2022). Likewise, there exists considerable literature on the topic of language acquisition in general, but when applied to differences among the dialects of China, the topic is less widely explored, and there appears a gap in the current research on this topic. While the topic of language and dialect acquisition, in general, is broad and is outside the scope of this study, a limited review of the existing literature is appropriate as part of the hypothesis development process.

This study relies on the work of Schepens et al. (2020) who use big data analytics to convincing report that linguistic similarities between L1 and L2 would have a significant effect on L2 proficiency, and conversely, dissimilarities in phonological, morphological and lexical measures result in lower proficiency in L2. These findings are consistent with previous research including Brown (1998) who reported that, in contrast with the theory of Universal Grammar (Mendívil-Giró, 2018), differences in phonemic contrasts may be undetectable to learners if their L1 does not contrast those segments. In other words, acquisition of L2 depends in part on the similarly between L1 and L2.

Given the range of diversity of these measures in the dialects discussed in see section “Chinese dialects – regions,” it follows that native speakers of the dialects would likely have differing levels of English language proficiency.

The diverse nature of the distinct dialects of China may affect the optimal ways for Chinese to learn English (Feng and Kim, 2022), which combined with variations in education among regions (Qian and Smyth, 2008; Tsung and Cruickshank, 2009) results in differing levels of proficiency amongst speakers of the different dialects These factors combined make it likely that the learners’ native dialect will affect their English language proficiency; therefore, this study hypothesizes that learners from different regions, represented through their regional dialects, would have differing educational propensities in learning English.

### Access to English language education

China’s economic reforms began in the late 1970s with the opening up of international trade and cross-border interaction, and it has changed China’s economy and social and cultural development. In the second half of the 1990s, realizing the importance of higher education in a knowledge-based economy, China began to expand higher education to meet the learning requirement of citizens (Gao, 2018; Gleason, 2018). Since 1998, China’s higher education has experienced three development processes: popularization, diversification, and internationalization, and the higher education system has undergone significant changes from the elite model to the popular model of popularization and internationalization (Gao, 2018; Mok and Marginson, 2021).

To support the goals of economic development based in part on technological innovation, English language proficiency is important. For students, specifically, those who want to go abroad to study, English test scores have become the most significant hurdle for gaining opportunities to study abroad (Jin and Yang, 2018; Reynolds and Teng, 2019). The complexities of English language acquisition have been explored and found to be an essential aspect of English language education (Wang and Jin, 2022). For example, the TOEFL (Test of English as Foreign Language) is the most popular test taken in the world, taken by 27 million people (Halim and Ardiningtyas, 2018), and preparation for taking the test has taken on immense importance for many young Chinese (Zheng et al., 2021). Chinese students who wish to attend a highly ranked university abroad must get a sufficiently high score on the TOEFL test.

A significant hurdle to the success of English language learning in China is the combination of a dramatic increase in the number of students seeking to learn English (Hu and Feng, 2012), the unfilled demand for Chinese English teachers (Wang and Zhang, 2021) and the inconsistent quality and access to English language education (Qian and Smyth, 2008; Cheng, 2009) while at the same time, the roles and expectations of ESL teachers is becoming more complex (Chen et al., 2022).

This hurdle disproportionately affects students in those less economically developed regions of China. Further, even in areas where economic development is advanced, there are variances in the type of development and the resulting level of emphasis placed on English language education. Given the differing access to English language education, it is likely that English proficiency would vary amongst the speakers of the dialects of those regions.

### Regional economic development

Since the economic reforms of the 1980s, China has pursued a policy of transformation to drive economic growth with a stated goal of poverty reduction (Yan, 2018), successfully achieving that goal in 2020. One of the initiatives designated as part of this effort is the emergent Industry 4.0, which has been widely mentioned and discussed in China. It refers to “the fourth industrial revolution” (Thames and Schaefer, 2016; Kuo et al., 2019). Industry 4.0 aims to change lifestyles, create new business and manufacturing models, update industrial structures based on environmental protection and control, increase resource use efficiency, encourage intelligent and flexible processes, and promote modern technology (de Sousa Jabbour et al., 2018; Müller and Voigt, 2018; Lin et al., 2019). Across multiple sectors, industries struggle to adapt to emerging models based on technological advances with industry 4.0 resulting from the normal competitive phenomenon in the economic development cycle, with many firms grappling with intelligence and digital transformation (Hou et al., 2020; Zhou et al., 2020).

There are two implications to this focused strategy for technology-driven economic development; the first is that there is a compelling need for a highly educated and technologically advanced workforce making advanced levels of education both important and competitive and resulting in increased interest in higher education and development of advanced technical skills.

The second implication is that technology-driven economic development has tended to take place inside of industry clusters (Huang and Wang, 2018), similar to Silicon Valley in the US (Etzkowitz, 2019). This tends to concentrate commercial development in certain areas resulting in higher investment, more international interaction, and greater benefits for the more highly educated members of the workforce, including greater allocation of resources to education in those areas (Bhawsar and Chattopadhyay, 2018).

Commercial development across sectors also shows a range of factors. For example, in some industrial cities, commerce is domestic B2B (Business to Business) or domestic B2C (Business to Consumer), (Wei et al., 2020), which does not have reliance on English proficiency, while in other sectors, commerce is primarily for export or Original Equipment Manufacturing (OEM) with more reliance on English communication with customers abroad (Hong, 2018; Kroll and Neuhäusler, 2020). Based on the combination of research regarding the different regional economic development models, it seems likely that the motivation for English language acquisition will differ among learners based on the regions where they are from.

### Foreign interaction

Historically, some areas in China have a long history of interaction and presence of foreigners. Shanghai, for example, is known for having experienced a significant influence from outside China over a period of several centuries as a trading center and with a high number of foreigners visiting and residing in the city (Maclellan, 1889; Guan et al., 2014). It is reported that a quarter of the foreigners residing in mainland China live in Shanghai (Farrer, 2019; Pieke et al., 2019), comprising about 1% of the city’s population (Pieke et al., 2019).

A noticeable trend in recent years is a growing number of non-native English speaking foreigners mostly Koreans and Japanese, but increasingly from other countries (Pieke et al., 2019). The implication is that those foreigners tend to rely heavily on English as the common language for commercial activity (Pieke et al., 2019). These trends play an important role in making Shanghai one of the world’s most cosmopolitan cities. The relatively higher level of interaction with foreigners over time has had considerable influence on the historical development of the city (De Giorgi, 2017), and is likely to have increased the number of English speakers in Shanghai, as well as had an effect on the type of motivation of Shanghainese to learn English, including for tourism and social purposes.

Similarly, Hong Kong, the principle city in the Cantonese speaking region has had centuries of interaction with foreigners (Abbas, 2000), and has experienced a high level of economic development over an extended period of time (Wong, 2002).

Each of the five regions and their associated dialects has had a distinct economic development and relationship with foreign influences. Some parts of China experience large numbers of foreign tourists (pre-COVID), while some areas have very little tourism.

### Motivation

The word motivation is derived from the Latin verb *movere*, which means “to move,” and it means what moves a person to make certain choices. Motivation is considered an interesting and popular topic for research, but also to be difficult because, as a concept, it has too broad a meaning (Dörnyei and Ushioda, 2013).

Motivation can be considered in two ways. Firstly, by the level, or intensity of the motivation, and secondly, by the type or reason for the motivation (Yashima, 2000). In this study, the focus is on the reason or cause of the motivation.

According to the seminal work of Krashen and the widely known and accepted (Jegerski, 2021) five hypotheses of his theory of second language acquisition, the Affective Filter hypothesis plays a key role in language acquisition (Chen, 2022). The Affective Filter, as a theoretical construct in second language acquisition attempts to explain the role of emotional variables in language acquisition. As such, the Affective Filter Hypothesis captures the relationship between those emotional variables and the process of language acquisition (Krashen, 1981). One way to describe the output of those variables is motivation (Chen, 2022), with the consequence that learners will differ from each other by the levels and types of emotions affecting the language acquisition process, and how they are filtered by each individual. As a result, learners will have differing levels and types, or reasons, for their motivation to acquire a second language. This study will build upon and expand Krashen’s Theory of Second Language Acquisition by applying the Affective Filter hypothesis to English language learners of different Chinese dialects.

Motivation is a crucial part of language learning (Dörnyei and Ushioda, 2013; Baxriddinovna and Guzaloy, 2022). In language learning, motivation can be the reason why we learn a foreign language or to what degree we work hard during the learning process. Motivation plays a major role in the results of language learning. While there are personal characteristics for each learner, L2 learners with high motivation are typically more likely to achieve positive results (Mahmoodi and Yousefi, 2022).

During the learning progress, learners may have different reasons for learning motivation. Altiner (2018) reported that the learner’s attitude toward the culture of L2 would affect their motivation. Polat (2020) found that in situations where learning L2 was related to their success in life, learners who experienced compulsory language education were not less motivated than those who chose it willingly and got similarly high test scores.

From a social psychology perspective, the early research of Gardner and Lambert (1959) determined the two primary factors for L2 learning as aptitude and motivation. Later research by Gardner (Gardner and Lambert, 1959) added one more factor and found three major types of orientations, aptitude, motivation, and method of presentation, finding the integrative orientation was highly important. Motivation which is integrative-centered is related to interest in the culture and language learning, whereas learners with instrumental-centered motivation is when the learner is focused on the practical purpose of learning L2, which was found to result in high proficiency scores as well (Lukmani, 1972; Jahan Khan et al., 2016).

Moreover, motivation is also classified into extrinsic motivation as well as intrinsic motivation based on the theory of self-determination (Deci and Ryan, 2000). Extrinsic motivation is when learners want to study for awards and recognition, while intrinsic motivation has less to do with the language itself and is closely associated with the needs of L2 learners themselves (Lepper et al., 2005), such as the requirement for study abroad, the desire to advance career and employment opportunities (She et al., 2008) or simply their personal interest in language learning (Hidayati and Diana, 2019).

While the educational system in China has developed rapidly, the perception, not without empirical evidence (Liu et al., 2021), remains that western higher education will provide Chinese graduates with enhanced opportunities, and this is a powerful incentive for Chinese to study abroad, which can be a powerful motivator for achieving the required English proficiency.

Based on these findings, it seems likely that different types of motivation will affect L2 (English, in the case of this study) proficiency.

## Conceptual framework-hypothesis development

This study focuses on Chinese learners’ proficiency in English as the dependent variable and their prior language/dialect acquisition as the independent variable. For this study, the dialect that the learners use most at home and with family would be their “most exposed dialect” (MED) and considered to be their first dialect (D1).

Based on the above review of existing literature on related topics, and to address the research questions, the following hypotheses are put forth for testing.

As discussed in see section “access to English language education,” the literature on the relationship between economic development and the resultant allocation of resources toward improving the quality of education, and the role of better quality education in improving English language learning and proficiency suggests that speakers of dialects from more economically developed regions are like to have access to better education and therefore achieve higher levels of English proficiency.

A review of the literature in see section “Chinese dialects – regions” indicates considerable differences between the linguistic characteristics of the dialects. These differences, when combined with the review of the literature regarding language acquisition amongst speakers of differing languages in see section “language acquisition,” which confirms that the differing linguistic characteristics between L1 and L2 will affect the learners’ proficiency in L2, make it reasonable to expect that speakers of these dialects will have differing propensities for English language acquisition Therefore, hypothesis 1 below is put forth for testing;

H1 English proficiency will differ by native dialect

Based on the application of Krashen’s Theory of Second Language Acquisition combined with the review of related studies on how motivation affects language acquisition discussed in see section “motivation,” it seems likely that different types, or reasons for learning English will affect English proficiency. Therefore, the following hypothesis is put forth for testing;

H2 Type of motivation will affect English proficiency

Likewise based in part upon the application of Krashen’s Theory of Second Language Acquisition, where the learners’ motivation will vary depending on their situation, combined with the findings regarding the different levels and types of economic development among the regions of China described in see section “regional economic development,” and the findings of related literature on the basis, duration and intensity of foreign interaction in the different regions discussed in see section “foreign interaction,” it seems likely that the motivating reasons for English language acquisition will differ among speakers of different native dialects and the regions where those dialects are spoken. Thus, this study puts forth the following;

H3 Type of motivation will differ by native dialect

Further application of the findings discussed in see section “motivation,” provides the basis for the development of the final hypothesis, that type of motivation will have a moderating effect on the relationship between MED and English proficiency. Hence, the following is put forth for testing;

H4 Type of motivation will moderate the effect of dialect on English proficiency differently among dialects.

To provide meaningful findings for the RQs, it becomes necessary to test each hypothesis separately for each dialect, and for each type of motivation, resulting in several analytical procedures for each. Hence, the hypotheses are expanded to;

H1a-e English proficiency will differ by native dialect

H2a-c Type of motivation will affect English proficiency

H3a-e Type of motivation will differ by native dialect

H4 Type of motivation will moderate the effect of dialect on English proficiency

The basis for identifying the types of motivation was derived from previous work and adapted for application in this study. The foundation for identifying social, professional/career, and educational motivation as the most suitable types of motivation was drawn from the findings of the work of Zheng et al. (2019), Dörnyei (2020), and Mahmoodi and Yousefi (2022).

**FIGURE 2:**
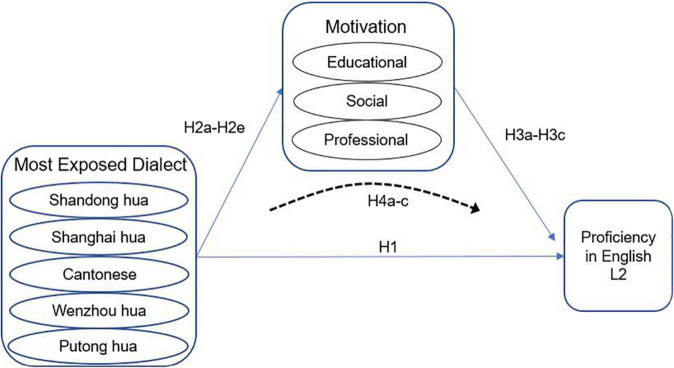
Theoretical framework.

The Conceptual Framework for this study is represented in Figure 2.

## Methodology

### Sample and data collection

This research analyzed quantitative data from an online survey to explore the research question, investigating the relationships among students’ proficiency in English, proficiency in different regional dialects, and motivation to learn English. The survey link was distributed through the social media platform WeChat. The data was compiled and downloaded via the Wen Juan Xing software application, where the survey platform was hosted, and the data was compiled.

For the purposes of this study, five of the primary dialects of China were chosen with the intent of providing a diversity of economically developed bases, urban to non-urban populations, and geographic areas. The dialects chosen are Shanghainese, Putong hua, Shandong, Cantonese, and Wenzhounese. A total of 1,203 respondents completed the survey. Subsequently, 12 participants were dropped due to irregularities in their responses, typically incomplete surveys. Participants from other regions of China, or who reported their native dialect as “other,” numbered 206 and were not included in the hypothesis testing where dialect was specified because their most exposed dialect was outside the scope of this study, leaving 985 respondents included in the analysis. Participants were informed that their responses would remain confidential and anonymous and of the voluntary nature of the survey.

Table 1 shows the demographic detail. Respondents included 617 females and 574 males. Nearly 32.6% of them have completed a 4-year University or College, which is relevant as English education has been required in China for many years.

**TABLE 1:** Demographics of the sample.

Characteristic		Frequency	Percentage
Gender	Male	574	48.2%
	Female	617	51.8%
Level of education	Completed high school	301	25.3%
	Completed vocational technology training	255	21.4%
	Presently enrolled in University/College	125	10.5%
	Completed 4 year University/College	388	32.6%
	Completed masters	122	10.2%
Local/regional dialect Note: *N* = 1,191	Shandong hua	185	15.5%
	Shanghai hua	229	19.2%
	Cantonese	203	17.0%
	Wenzhou hua	222	18.6%
	Beijing hua	146	12.3%
	Other (not included)	206	17.3%

More than half of them rated their proficiency in their respective regional dialects as high. Surprisingly, they are quite confident in their English proficiency also, a finding which was relatively evenly distributed across different segments of the sample.

### Instrumentation

Previous used and validated measurement tools and scales were applied to measure the independent, dependent, and moderating variables. Students’ proficiency in English was measured by Likert scale questions adapted from Matell and Jacoby (1971). For the instrument weighing variables, a seven-point Likert scale ranging from “1” (strongly disagree) to “7” (strongly agree) was used. A seven-point scale is a recognized tool that provides optimal reliability (Allen and Seaman, 2007; Joshi et al., 2015).

#### Native dialect

The dimension of the language used by individuals in different environments was based on the everyday environments where learners typically live. While it may appear evident that most would speak Mandarin at school, it is possible that in some regions, other dialects may be spoken in schools or at the workplace:

• What is your local/regional dialect?
• What dialect do you speak at home?
• What dialect do you speak with your family?
• What dialect is most spoken in your hometown?

#### English language proficiency

The dependent variable, language proficiency in English, was ascertained from survey items adapted from previous research (Wang and Jin, 2022). The description is as follows, “I can complete basic forms and write notes including times, dates, and places.”, “I can write letters or make notes on familiar, predictable matters.”, “I can write letters on any subject and full notes of meetings or seminars with good expression and accuracy.”, “I can understand basic notices, instructions, or information.”, “I can scan written materials for relevant information and understand detailed instructions,” “I can understand documents, correspondence, and reports, including the finer points of complex texts.”

Additional survey items used to assess proficiency in the languages/dialects included questions validated in previous research (Manan and David, 2014) and adapted for this study. The description is as follows, “I can understand instructions and take part in basic conversations about familiar topics.”, “I can express my opinions and requirements in a familiar context.”, “I can conduct business and day-to-day transactions.”, “I can contribute to meetings and give advice on about complex or sensitive issues.”, “I can understand slang and colloquial references.”, “I can deal confidently with strangers who are native speakers of my dialect.”

#### Motivation for learning English (reason)

The dependent variable, motivation for learning English (reason), was adapted from previous research (Zheng et al., 2019) was validated and included multiple-choice questions; “I think that learning English would be useful to me for;” “I study English because;” “I plan to study English in the future for;” “I think that in the future, knowing English would be useful for.”

## Results

### Reliability analysis

Each of the constructs was evaluated for reliability using the accepted method of Cronbach’s testing (Boone and Boone, 2012). As shown in Table 2, all of the Cronbach’s α values range between 0.76 and 0.99, which is in the acceptable range (> 0.60) (Hair, 2009). This ensures that the constructs used in this research are reliable for further analysis.

**TABLE 2:** Cronbach validity.

Constructs	Cronbach alpha	*N* of items
Most exposed dialect	0.98	4
Mandarin proficiency	0.99	6
English proficiency	0.97	12
Educational reasons	0.96	8
Social reasons	0.99	12
Career reasons	0.76	5

To test the Hypotheses of this study, the data was subjected to Simple Regression Analysis, Multiple Regression Analysis, and Moderation Analysis.

### H1a-e English proficiency will differ by native dialect

H1 was tested by Analysis of Variance. A one-way between-groups analysis of variance (ANOVA) was used to investigate if the English language proficiency differs by most exposed dialect. As indicated in Table 3, there was a statistically significant difference in Proficiency in English scores for the five dialect groups, *F*(4,1084) = 226.78, *p* < 0.001.

**TABLE 3:** Proficiency by MED.

Most exposed dialect	*N*	Mean	Std. Deviation
Shandong hua	194	1.75	1.02
Shanghai hua	193	4.88	1.14
Cantonese	175	3.29	1.70
Wenzhou hua	207	2.09	1.44
Putong hua	320	1.72	1.17

Therefore, H1 is supported.

Further analysis reveals the specific difference between MEDs as indicated in Table 4 with Shanghainese and Cantonese showing notably higher levels of English proficiency, and Shandong hua speakers with lower levels. Unexpectedly, Putong hua speakers also show relatively low levels of English proficiency as is discussed in detail in see section “discussion.”

**TABLE 4:** Comparative English proficiency.

(I) Dialect	(J) Dialect	Mean difference (I-J)	Std. Error	Sig.	95% Confidence interval	
					Lower boundary	Upper boundary
Shandong hua	Shanghai hua	−3.13*	0.11	0.000	−3.43	−2.83
	Cantonese	−1.54*	0.15	0.000	−1.95	−1.13
	Wenzhou hua	−0.34*	0.12	0.047	−0.68	0.00
	Putong hua	0.02	0.10	0.999	−0.25	0.29
Shanghai hua	Shandong hua	3.13*	0.11	0.000	2.83	3.43
	Cantonese	1.59*	0.15	0.000	1.17	2.01
	Wenzhou hua	2.79*	0.13	0.000	2.43	3.14
	Putong hua	3.15*	0.10	0.000	2.87	3.44
Cantonese	Shandong hua	1.54*	0.15	0.000	1.13	1.95
	Shanghai hua	−1.59*	0.15	0.000	−2.01	−1.17
	Wenzhou hua	1.20*	0.16	0.000	0.75	1.64
	Putong hua	1.56*	0.14	0.000	1.17	1.96
Wenzhou hua	Shandong hua	0.34*	0.12	0.047	0.00	0.68
	Shanghai hua	−2.79*	0.13	0.000	−3.14	−2.43
	Cantonese	−1.20*	0.16	0.000	−1.64	−0.75
	Putong hua	0.37*	0.12	0.020	0.04	0.69
Putong hua	Shandong hua	−0.02	0.10	0.999	−0.29	0.25
	Shanghai hua	−3.15*	0.10	0.000	−3.44	−2.87
	Cantonese	−1.56*	0.14	0.000	−1.96	−1.17
	Wenzhou hua	−0.37*	0.12	0.020	−0.69	−0.04

*The mean difference is significant at the 0.05 level.

### H2 type of motivation will differ by native dialect

H2 was tested by one-way between-groups analysis of variances (ANOVA) to determine if the Educational, Social, and Professional motivation types differed by MEDs. Each motivation type was tested separately.

#### Educational motivation

As indicated in Table 5, there was a statistically significant difference in educational motivation among the five dialect groups, *F*(4,1084) = 568.05, *p* < 0.001.

**TABLE 5:** Educational motivation by most exposed dialect.

	*N*	Mean	Std. deviation
Shandong hua	194	2.05	0.92
Shanghai hua	193	5.28	0.60
Cantonese	175	5.23	0.60
Wenzhou hua	207	1.99	0.69
Putong hua	320	2.06	1.58

*Post hoc* comparison indicated that respondents speaking Shandong hua have significantly lower educational motivation compared to Shanghai hua and Cantonese. Both Shanghai hua and Cantonese have significantly higher educational motivation compared to Shandong hua, Wenzhou hua, and Putong hua. Wenzhou hua has significantly lower educational motivation compared to Shanghai hua and Cantonese. Putong hua has significantly lower educational motivation compared to Shanghai hua and Cantonese.

#### Social motivation

As indicated in Table 6, There was a statistically significant difference in social motivation among the five dialect groups, *F*(4,1084) = 1400.41, *p* < 0.001.

**TABLE 6:** Social motivation by MED.

	*N*	Mean	Std. deviation
Shandong hua	194	1.39	1.02
Shanghai hua	193	5.42	0.54
Cantonese	175	5.31	0.71
Wenzhou hua	207	1.45	0.76
Putong hua	320	1.44	0.91

*Post hoc* comparison indicated that respondents speaking Shandong hua have significantly lower social motivation compared to Shanghai hua and Cantonese. Both Shanghai hua and Cantonese have significantly higher social motivation compared to Shandong hua, Wenzhou hua, and Putong hua. Wenzhou hua has significantly lower social motivation compared to Shanghai hua and Cantonese. Putong hua has significantly lower social motivation compared to Shanghai hua and Cantonese.

#### Professional motivation

As shown in Table 7, there was a statistically significant difference in Professional motivation among the five dialect groups, *F*(4,1084) = 57.19, *p* < 0.001.

**TABLE 7:** Professional motivation by MED.

	*N*	Mean	Std. deviation
Shandong hua	194	4.27	1.26
Shanghai hua	193	5.73	0.82
Cantonese	175	4.37	1.25
Wenzhou hua	207	4.40	1.19
Putong hua	320	4.33	1.22

*Post hoc* comparison indicated that respondents speaking Shandong hua have significantly lower Professional motivation compared to Shanghai hua. Shanghai hua has significantly higher Professional motivation compared to Shandong hua, Cantonese, Wenzhou hua, and Putong hua. Wenzhou hua has significantly lower Professional motivation compared to Shanghai hua. Putong hua has significantly lower social motivation compared to Shanghai hua.

### H3a-c type of motivation will affect English proficiency

Multiple linear regression analysis was used to investigate the effect of three different types of motivations on English language proficiency. In combination, educational, social, and Professional motivations predict 40% of the variance in English language proficiency, *R*^2^ = 0.40, *F*(3,1107) = 244.53, *p* < 0.001.

Educational motivation has a significant effect on English language proficiency (Beta = 0.10, *p* = 02). The coefficient of educational motivation indicates that if educational motivation increases by 1 unit, the English language proficiency will be increased by 0.10 units, holding other variables constant. Therefore, H3a is supported.

Social motivation has a significant effect on English language proficiency (Beta = 0.40, *p* < 0.001). The coefficient of social motivation indicates that if social motivation increases by 1 unit, the English language proficiency will be increased by 0.40 units, holding other variables constant. Therefore, H3b is supported.

Professional motivation has a significant effect on English language proficiency (Beta = 0.18, *p* < 0.001). The coefficient of Professional motivation indicates that if Professional motivation increases by 1 unit, the English language proficiency will be increased by 0.18 units, holding other variables constant. Therefore, H3c is supported.

Each of the three types of motivation has a significant effect on English proficiency; therefore, all three H3 hypotheses are supported.

### H4 motivation type will moderate English proficiency differently by most exposed dialect

While the finding that H3a-c Type of motivation will affect English proficiency is supported is not without relevance on its own, that finding fulfills an important foundational role in the development of H4, Type of motivation will moderate the effect of MED on English proficiency differently by MED. H4 is the primary test to address the research question and the determinant of the value of the contribution made by this study.

As indicated in Table 8, MED and Educational motivation accounted for 47% variance in English language proficiency, *F*(9,1079) = 106.55, *p* < 0.001.

**TABLE 8:** Moderation effect of educational motivation.

	Coefficient	*P*
Constant	0.80	0.000
Shanghai hua	3.67	0.000
Cantonese	2.13	0.015
Wenzhou hua	1.00	0.004
Putong hua	0.67	0.008
Education	0.46	0.000
Shanghai hua Edu	−0.38	0.035
Cantonese Edu	−0.39	0.037
Wenzhou hua Edu	−0.32	0.049
Putong hua Edu	−0.34	0.002

There is a significant moderation effect of educational motivation on the relationship between MEDs and English language proficiency.

As indicated in Table 9, Dialect and Social motivation accounted for 48% of the variance in English language proficiency, *F*(9,1079) = 110.42, *p* < 0.001. Social motivation has a significant moderating effect on the relationship between MEDs and English proficiency.

**TABLE 9:** Moderation effect of social motivation.

	Coefficient	*P*
Constant	1.16	0.000
Shanghai hua	2.05	0.029
Cantonese	1.03	0.165
Wenzhou hua	0.90	0.000
Putong hua	0.05	0.809
Education	0.43	0.000
Shanghai hua Edu	−0.12	0.542
Cantonese Edu	−0.22	0.180
Wenzhou hua Edu	−0.40	0.006
Putong hua Edu	−0.07	0.572

As indicted in Table 10, Dialect and Professional motivation accounted for 46% variance in English language proficiency, *F*(9,1079) = 101.88, *p* < 0.001. Professional motivation does not have a significant moderating effect on the relationship between MEDs and English proficiency.

**TABLE 10:** Moderation effect of professional motivation.

	Coefficient	*P*
Constant	1.93	0.000
Shanghai hua	2.35	0.001
Cantonese	0.75	0.121
Wenzhou hua	−0.45	0.343
Putong hua	−0.14	0.745
Education	−0.04	0.552
Shanghai hua Edu	0.15	0.286
Cantonese Edu	0.18	0.094
Wenzhou hua Edu	0.18	0.096
Putong hua Edu	0.03	0.785

The premise of H4 is that speakers of different dialects, representing different regions and circumstances would be motivated by different types of motivation. The analysis indicates that both Social and Educational motivation types do have a significant moderation effect on English proficiency; learners overall are not highly motivated by professional reasons, that is, to improve their career prospects. Therefore, Ha and Hb are supported, while H4c is not supported.

As indicated in Table 11, the hypotheses formulated on differing proficiencies among MEDs, the importance of motivation as a driver of English proficiency, and differing types of motivation among MED speakers are supported. The moderating effect of moderation on English proficiency is only supported for Social and Educational motivation, while Professional motivation does not have an effect on the relationship between native dialect and English proficiency.

**TABLE 11:** Hypothesis testing summary.

Hypothesis	Status
H1a-e English proficiency will differ by native dialect	Supported
H2a educational motivation will differ by native dialect	Supported
H2b social motivation will differ by native dialect	Supported
H2c professional motivation will differ by native dialect	Supported
H3a-c type of motivation will affect English proficiency	Supported
H4a educational motivation will moderate the effect of dialect on English proficiency	Supported
H4b social motivation will moderate the effect of dialect on English proficiency	Supported
H4c professional motivation will moderate the effect of dialect on English proficiency	Not supported

## Discussion

This study examined data gathered from 985 responses to an online survey distributed via social media in China. The distribution of respondents across the five target demographic groups, different native dialect speakers, was effective, and all groups were represented in the findings. The use of native dialects in China is common, and there are dozens, each spoken in a different geographic area, sometimes several versions within one large city. This results in the possibility of a lack of distinctiveness in data and brings to light the complexity of studies of this type, which perhaps helps explain the relative under exploration of the topic.

Nevertheless, the findings are revealing. There is a relatively high level of conformity in the responses, as indicated by the findings of the Cronbach test for validity. This has been observed in previous research, with one possible explanation being the highly conformist nature of Chinese society (Marjerison et al., 2022).

There is a significant difference in English proficiency between the MED speakers with speakers of Shanghainese having by far the greatest proficiency. This can be explained in the hypothesis development section above as a likely combination of centuries of commerce related interaction with foreigners, including the more recent influence of relatively high numbers of foreign residents and tourists and a high level of international commercial activity combined with the relatively high levels of economic development which in turn results in both more access to education, and higher quality of education.

Cantonese speakers are the next most proficient speakers of English. This is consistent with the findings of Cheng (2009), likely for much the same reasons, close proximity to the highly economically developed major city of Hong Kong combined with the resulting strong educational system, and centuries long exposure to foreigners and British influence (Abbas, 2000).

Wenzhou hua is the next highest level of English proficiency. While Wenzhou is not known for tourism, and has relatively few foreign residents, the findings can be explained by the findings of prior related research as the Wenzhou metro area is well known as a commercial hub for light manufacturing which results in the need for some English proficiency to support the exporting of products to Western markets (Newman et al., 2007) and for a high level of foreign cultural exchange (Cao, 2010).

Finally, Putong hua and Shandong hua have the lowest levels of English proficiency. In the case of Shandong hua the findings are predictable as the area has less interaction with foreigners historically, and presently experiences fewer foreign tourists than either Shanghai or Beijing.

In the case of Putong hua, the dialect of Beijing, the findings are less obviously explained. Certainly the capital has economic development and the accompanying resources allocated to education and has experienced both high levels of tourism and commercial development in recent years. There is a difference, however, between Beijing and the Shanghainese and Cantonese speaking regions when it comes to the level of foreign interaction over an extended period of time, as well as the sheer number of foreigners residing in those areas in recent decades.

The findings regarding the type of motivation show a similar pattern with Shanghai hua and Cantonese speakers much more likely to be motivated for educational purposes which is consistent with the findings of Liu and Chiang (2019). The explanation for this distinction is likely similar to that regarding the findings related to proficiency. As reported in the literature, for speakers of those two dialects, English language education is widely available, and of relatively high quality. The higher proficiency levels will also serve as motivational in the educational setting.

Shandong hua, Wenzhou hua and Putong hua speakers have nearly the same level of educational based motivation. Similarly, to the findings for H1 Proficiency, Shandong hua and Wenzhou hua speakers’ lower motivation based on educational purposes is understandable. The case of Beijing, however, is less predictable. Notably, Beijing is home to several highly regarded Chinese language medium universities which could be a factor.

Shanghai hua and Cantonese speakers are much more likely to be motivated by social factors than speakers of the other three dialects. These findings are compatible with those discussed above and add the implication that these two regions have some social activities that are English language based due to interaction with foreign residents can be explained by the higher numbers of foreign residents in the major cities of Hong Kong and Shanghai. Wenzhou and Shandong have relatively few foreign residents so motivation to learn English for social purposes is understandably low. Again, the Putong hua speakers, many of whom are residents of the capital city of Beijing, are not motivated to learn English for social purposes. The implication is that while Beijing is a major city, with many foreign residents, a high level of tourism, and highly economically developed, there is less interest in social activities with non-Chinese speakers. There may be geopolitical and cultural implications to this finding due to differing cultural perceptions with regards to the desirability of a cosmopolitan environment. This finding could contribute to the consideration of social, geopolitical, and cultural influences on language acquisition. When it comes to motivation based on Professional purposes, for career development, Shanghai alone stands out with the other four showing relatively equal and significantly lower levels of motivation. The implication here is that English language proficiency is perceived as enhancing one’s career and employment prospects in Shanghai to a much greater extent than in the other regions. This can be explained in part by the findings of prior research that reported that while other regions may be highly developed commercially, and have many foreign residents and tourists, Shanghai is known as a major finance center as well as a gateway for commerce between China and other nations (Abbas, 2000; Wong, 2002).

The moderation effect of motivation type on the relationship between native dialect and English proficiency is significant for motivation based on educational purposes for all respondents combined, but most highly in the case of Shanghai hua speakers with Cantonese speakers second. Speakers of the other three dialects do not show increased proficiency as a result of education-based motivation. Likewise, and consistent with the findings of the other hypothesis testing, motivation for social purposes shows a moderating effect while motivation for professional purposes does not have a significant influence on the relationship between dialect and English proficiency. The implication of these findings on the moderating effect of the type of motivation on English proficiency indicates that English language acquisition for career and professional advancement purposes is not a strong driver, while both social purposes, and especially educationally related purposes are strong drivers of English proficiency.

### Theoretical contribution

The findings of H1a-e English proficiency will differ by native dialect, present three theoretical contributions to the existing research. Firstly, in the areas of linguistics, the findings extend the theories of language acquisition discussed in see section “language acquisition” by building on the findings of existing theories of differing propensities for language acquisition based on a variety of factors including linguistic and cultural similarities and perceptions. This study addresses a gap on how differing native Chinese dialects can have an effect on English language acquisition.

Secondly, in the area of sustainable economic development, an additional theoretical contribution can be identified in the elevated level of English language proficiency in the coastal economic and financial center of Shanghai, while the diplomatic and capital city of Beijing has a noticeably lower level of English proficiency. These findings suggest that differing types of economic development activities may have an effect on English language acquisition, and by extension, on access to the higher education, which is a precursor to socioeconomic mobility. The findings provide empirical evidence that those whose most exposed dialect is Shanghai hua have a distinctly higher proficiency in English. This is likely a result of a combination of foreign tourism, commerce, and the centuries of foreign interaction.

A final theoretical contribution can be found in the area of motivational theory on language acquisition. This study builds upon and expands Krashen’s Theory of Second Language Acquisition by introducing the variable of type of motivation to the exploration of English language proficiency among native speakers of different dialects of China. Other existing theories, as discussed in see section “motivation” (Altiner, 2018) make clear the importance of motivation in language acquisition, however, a gap exists in how differing types of motivation, as defined in this study based on previous research, may affect English language acquisition by native speakers of different major dialects of China. H2a-e the Type of motivation will differ by native dialect combined with H3a-c Type of motivation will affect English proficiency builds directly on and extends the prior research discussed above (Baxriddinovna and Guzaloy, 2022) as well as the results of H1 by suggesting an possible explanation for the differing levels of English proficiency based on motivation. These findings are consistent with results reported by Polat (2020) and contributes to the theories of motivation, and motivation for language acquisition discussed in see section “motivation” including by extending the previous findings to include speakers of different native dialects in China, and by extension, to the different cultural and economically developed regions from which they come. Interestingly, Beijing, even as the capital city with links to substantial numbers of foreigners through both diplomatic and tourism activity, who most commonly use English as the language of interaction, has distinctly lower levels of English proficiency.

Additionally, H3 contributes to the literature on how different types of motivation impact language acquisition differently. While all three types examined in this study, social, educational access, and professional advancement related, were all found to be motivational in English language acquisition, motivation remains an important area of research, especially in language acquisition, the importance of English proficiency as a motivator for those seeking to improve the economic circumstances is important. Mapping these findings into similar research on motivation may lead to insights into motivational theories in language acquisition.

### Practical contribution

There are practical contributions to be derived from this study. With the aim of ascertaining how better to drive economic prosperity through access to education, and given the importance of English proficiency as a driver of access to higher education, some relevant observations can be made.

The differing types of motivation among the speakers of the dialects and the regions they represent also present an interesting outcome for those interested in development planning. The stated goal of the Chinese government, economic prosperity through education, may entail the allocation of additional and specific types of resources to support educational initiatives in different regions.

Depending on the type of commercial and cultural activity in a region or a city, learners will focus on differing reasons for English language acquisition. As discussed in the literature review and hypothesis development sections, those regions which have had a higher level of interaction with foreigners either through trade or, more recently, through tourism are more motivated by social opportunities. Dialects where there is less foreign interaction are less motivated by social reasons, but are more likely to be motivated by for professional reasons. Consideration of the type of motivation may be relevant for educators, specifically those focused on language acquisition, in tailoring the educational programming in different regions.

Finally, the findings related to language acquisition specifically within China and for those with an interest in different types of motivation are relevant to linguists and educators focused on language acquisition.

## Conclusion

This study provides both practical and theoretical contributions to the existing literature in a relatively under-examined area of inquiry, as reviewed in detail in see section “discussion” above. This research is motivated by the premise that a sustainable society includes addressing and reducing social problems, including, through enhancing opportunities for socioeconomic mobility, which can be brought about through access to education. Because English language proficiency is the major hurdle for Chinese students seeking to study abroad, and because of the economic advantages that result from international higher education, it is relevant to examine how students of different socioeconomic backgrounds may have different propensities toward English language acquisition.

Additionally, this article makes the case that speakers of some dialects, which may be from less economically developed areas, are at a competitive disadvantage in competing for education opportunities abroad and that English proficiency is perceived as a way for less advantaged students to achieve career success, and by extension, improve their economic circumstances. This study also makes the case that while the level of intensity of motivation is an important aspect of language acquisition, different reasons for motivation also play an important role.

Therefore, English language education should be both encouraged and supported in regions where English proficiency is lower, and English language education should be tailored specifically to meet the developmental styles of speakers of different native Chinese dialects in order to support the achievement of the stated goal of a sustainable society based on socioeconomic mobility through access to education.

## Limitations and future research

No research is without limitations and in the case of this study, sample size is one. Replicative research on a larger sample size would strengthen the findings. The age range of the respondents to the online survey could make the supported findings more specific and possibly more relevant from one perspective. Further examination of the data from this study or from further research on the same or a similar topic, including for example detailed pairwise comparisons could provide additional insights.

The five regions from which native speakers were included in this study are not representative of all of China. Future research of a similar nature that focused on other dialects from other regions would provide interesting insights into the broad diversity of dialects, as well other circumstances not examined in this study.

This study found correlations between English language proficiency and two dialects, Shanghai Hua and Cantonese, while providing more than one possible explanation for the correlation. This is both a shortcoming and an opportunity for future research. Determining whether that correlation results from social and culture factors, historical interaction with foreigners, or from the quality and focus of the educational system would be a valuable contribution for future research to which this work can provide a foundation.

The unexpected finding of lower English language proficiency in the capital city of Beijing is worthy of further examination. While possible explanations for this finding, based on the existing literature are provided above, there are other considerations. The samples used in this study, particularly in the case of the Beijing dialect, may not be representative of the population due to shortcomings in the nature of a self-selecting online survey. Additionally, respondents’ proficiency was measured though their perceptions of their own proficiency, which could be affected by their perceived proficiency relative to their peers which could have a confounding effect on the results. Further examination of this finding is warranted.

This study may provide a foundation for further studies on how different demographic groups may be provided opportunities for socioeconomic activity. For example, considerations of gender, specific background, proximity to individuals with different levels of education, and family income could all provide valuable insights.

While this study focuses on the motivation and proficiency of speakers of five of the major dialects of Chinese, similar research in other countries where there are multiple languages/dialects spoken would provide valuable insights, and if results were similar, increase both the value and generalizability of this study. Finally, a related study on outcomes or results of English proficiency would be interesting to validate the widely held perception that English is a means of socioeconomic mobility.

## Data availability statement

The raw data supporting the conclusions of this article will be made available by the authors, without undue reservation.

## Ethics statement

Ethical review and approval was not required for the study on human participants in accordance with the local legislation and institutional requirements. The patients/participants provided their written informed consent to participate in this study.

## Author contributions

SY: conceptualization and software. RM and SY: data curation and investigation. RM: formal analysis, supervision, writing – review and editing. Both authors have read and agreed to the published version of the manuscript.
